# An Esophageal Cooling Device During Radiofrequency Ablation for Atrial Fibrillation—A Comparison Between Reactive and Proactive Esophageal Cooling

**DOI:** 10.19102/icrm.2022.13111

**Published:** 2022-11-15

**Authors:** Brad Clark, Nazia Alvi, Joseph Hanks, Christopher Joseph, Brad Suprenant

**Affiliations:** ^1^St. Vincent Hospital, Indianapolis, IN, USA; ^2^Department of Cardiology, Riverside Medical Center, Kankakee, IL, USA; ^3^UT Southwestern Medical Center, Dallas, TX, USA

**Keywords:** Ablation, atrial fibrillation, esophageal cooling catheter, pulmonary vein isolation

## Abstract

Esophageal thermal injury is one of the most feared risks of ablation of the posterior left atrium despite the various devices used to monitor esophageal temperature or deviate the esophagus. Reactive cooling, in which cold water is manually instilled into the esophagus via an orogastric tube in response to rises in luminal esophageal temperature (LET), has been used by operators, but the availability of a dedicated esophageal cooling device offers the ability to provide proactive esophageal cooling without having to react to individual temperature rises in the esophagus. The objective of this study was to evaluate the feasibility of using a commercially available esophageal cooling device to provide esophageal protection during left atrial catheter ablation, then to compare this approach to standard LET monitoring with reactive cooling via manual cold-water instillation. In this study, we randomized 6 patients undergoing catheter ablation for atrial fibrillation. Three patients received the standard of care for our site (use of a single-sensor temperature probe, with adjunct ice-water instillation for any temperature increases of >1°C). Three patients underwent standard ablation after placement of the esophageal cooling device at a circulating water temperature of 4°C, without the use of any esophageal temperature monitoring. All patients underwent transesophageal echocardiography and esophagogastroduodenoscopy on the day prior to the ablation, followed by a second esophagogastroduodenoscopy the day after. The 6 patients in this study were enrolled between March and August 2018. In the 3 control patients, 1 had no evidence of esophageal mucosal damage, 1 had diffuse sloughing of the esophageal mucosa and multiple ulcerations, and 1 had a superficial ulcer with a large clot. Both patients with lesions were classified as 2a cases using the Zargar grading scheme for caustic injury. In the 3 patients treated with the cooling device, 1 had no evidence of esophageal mucosal damage, 1 had esophageal erythema (Zargar grade 1), and 1 had a solitary Zargar grade 2a lesion. At 3 months of follow-up, 1 patient in each group had recurrence of atrial fibrillation. Although a number of subsequent studies have confirmed the reduction of esophageal injury with the use of proactive esophageal cooling, this study is the only one to date to compare reactive cooling (via manual cold-water instillation) and proactive cooling (via a dedicated esophageal cooling device). Moreover, this is the first study to support the feasibility of using a dedicated cooling device for this purpose and provides the basis for further investigation.

## Introduction

The treatment of atrial fibrillation via pulmonary vein isolation using radiofrequency (RF) ablation has well-documented success. A rare complication of this procedure is atrio-esophageal fistula (AEF) formation.^1,2^ A precursor to AEF involves esophageal submucosal and possibly mucosal injury.^3^ This damage is caused by heating of the posterior aspect of the left atrium to create a transmural lesion that may cause further thermal injury to the esophageal tissue. Esophageal mucosal injury occurs in ≤30%–50% of patients undergoing ablation.^4^ A subset of these injuries, generally the higher-grade lesions, can then progress to AEF weeks after the initial injury.^5^

Various attempts to protect the esophagus have been evaluated, including the use of lower power settings, luminal esophageal temperature (LET) monitoring, mechanical displacement of the esophagus, and multiple cooling modalities, but few have shown clear clinical benefits. Meanwhile, cooling techniques that have been evaluated include reactive ice-water instillation, a cooling sac (EPSac; RossHart Technologies Inc., Cleveland, OH, USA), and various types of cooling balloons. Many of these approaches have been tested in bench models and animals and, in some cases, humans; until recently, however, no device had been available commercially.^6–13^

One commonly used approach to esophageal protection involves LET monitoring with adjustments made to the ablation location and timing based on temperature changes. However, the previously theorized efficacy of this approach has been demonstrated to be no better and in fact possibly worse than no temperature monitoring.^14,15^ Moreover, it has been suggested that the LET probe also contributes to thermal injury.^16^ The recently concluded mechanism for increased injury when relying on LET monitoring is the delayed notification, or the complete absence of detection, of dangerous temperature levels.^17,18^ As LET is representative of the degree of thermal insult from ablation after it passes through the posterior wall and all layers of the esophagus, the temperature alarms likely sound long after esophageal injury has already occurred.

**Figure 1:**
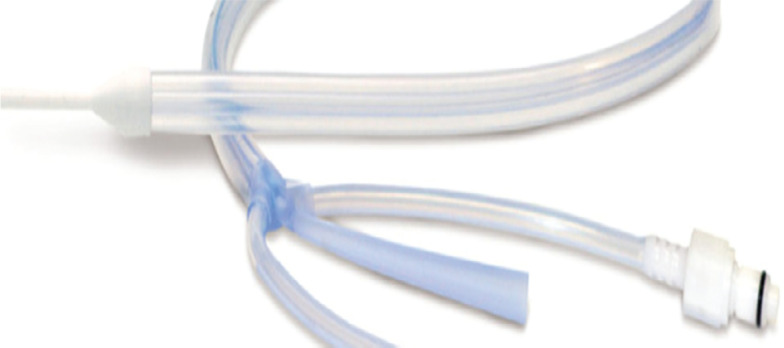
EnsoETM esophageal cooling catheter (Attune Medical, Chicago, IL, USA).

In 2015, a dedicated device for the management of patient temperature via the esophagus became available **(Figure 1)**. With a mechanism of action of direct cooling against the esophageal mucosa, we theorized that this device may provide a means of protecting the esophagus against thermal injury during RF ablation. For this reason, we performed the first study to determine the feasibility of utilizing this device in RF ablation, aiming to compare reactive cooling (performed via ice-water instillation into the esophagus through an orogastric tube in response to LET elevations) and proactive cooling with this commercially available esophageal cooling device.

## Methods

### Study population

Six adults diagnosed with an atrial arrhythmia were included in this blinded (patient and outcome assessor), randomized, controlled feasibility study. Patients considered for ablation of the posterior wall (non-pregnant, aged 18–90 years, and without prior esophageal damage or disease) were asked to participate in the study after providing written informed consent. The study was approved by the hospital’s institutional review board and registered at ClinicalTrials.gov under identifier #NCT03481023.

### Selection of participants

Permutated block randomization was used for group designation. The study included 1 female and 5 male subjects aged 55–71 years. Two patients had mild reductions in left ventricular ejection fraction (LVEF), with the remainder of the patients displaying normal LVEFs. Preprocedural diagnoses included paroxysmal atrial fibrillation (5/6), typical atrial flutter (1/6), and atypical atrial flutter (2/6). All patients had failed ≥1 anti-arrhythmic agent prior to the procedure. An outpatient visit was set for 3 months after ablation to assess for adverse events and to arrange for further evaluation of atrial arrhythmia recurrence **(Table 1)**.

### Measurements

Patients underwent transesophageal echocardiography (TEE) the day prior to the ablation, which was coupled with an endoscopic evaluation including an esophagogastroduodenoscopy (EGD). An independent gastroenterologist blinded to the patient’s assignment graded esophageal mucosal damage. We used Zargar’s modified endoscopic classification scheme to provide an objective quantification of mucosal changes **(Table 2)**. This classification scheme expands on the customary endoscopic classification of burns (grades 0–3) by subdividing grade 2 burns into grades 2a and 2b and grade 3 burns into grades 3a and 3b for prognostic and therapeutic implications.

All patients were observed in the hospital overnight with repeat EGD performed on the morning following the ablation procedure. Follow-up care included a 1- to 2-week assessment as well as a 3-month visit. All patients were evaluated via an event monitor or a loop recorder at between 3–6 months after ablation.

### Ablation parameters

All patients had general anesthesia as is customary for the study institution. All ablations were performed by the same electrophysiologist with the choice of ablation including any combination of pulmonary vein isolation, true left atrial roof line, mitral line, cavotricuspid isthmus, and complex fractionated atrial electrogram ablation as was clinically indicated. Control patients received a single-sensor temperature probe placed in the esophagus as well as a standard nasogastric tube placed to the depth of the left atrium. The temperature readings were monitored by laboratory staff during ablations of the posterior wall. In the event that the temperature rose by >1°C from baseline, ablation would cease, and the anesthesiologist was instructed to instill ice-cold water into the nasogastric tube in 10- to 20-mL aliquots. Ablation would then resume after equilibration of temperature readings to patient baseline. Ablation was carried out with 35 W of energy at 55°C on the posterior wall and 50 W of energy at 55°C on the anterior wall using a 4-mm-tip RF catheter. The RF catheters were non–contact-force sensing and non-irrigated.

### Intervention

The treatment group underwent the same preoperative assessment with TEE followed by EGD on the day prior to ablation, and their postoperative care and assessments were also identical to those of the control group. The treatment group patients underwent placement of a commercially available esophageal cooling device **(Figure 1)** into the esophagus. This device provides a closed-circuit flow of water through a multichannel cylindrical silicone tube placed in the esophagus analogously to a standard orogastric tube, heating or cooling a patient through conductive heat transfer across the esophagus and convective heat transfer through the device.^19^ The device was connected to a heat exchange unit (Blanketrol III Hyper-Hypothermia System; Gentherm Medical, Cincinnati, OH, USA), which allows for a large-volume flow of temperature-regulated distilled water at a rate of 136 L/h. Just prior to the ablation, the coolant was set to 4°C, and this temperature was maintained during ablation of the posterior wall. The esophageal temperature was not monitored in the treatment group.

## Results

### Characteristics of participants

A total of 6 patients were enrolled between March and August 2018.

### Endoscopy results

**Figure 2:**
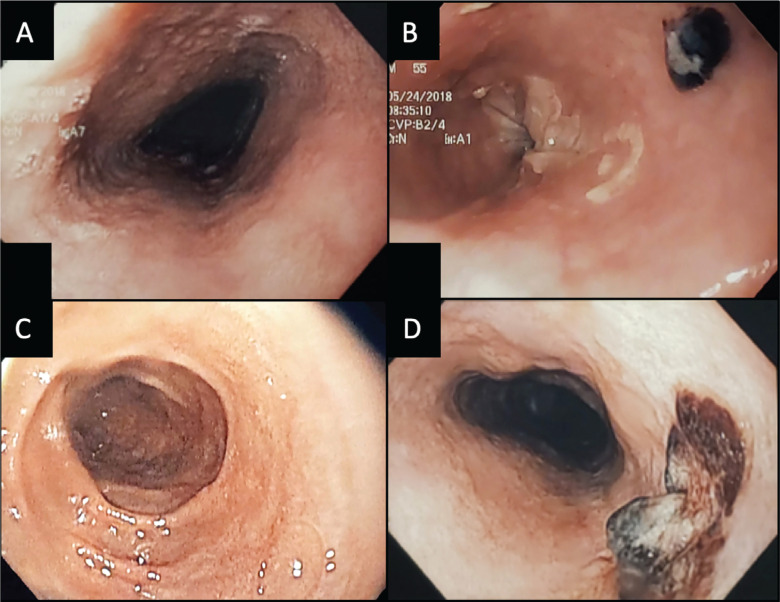
Esophagogastroduodenoscopy images of control patients showing esophageal ulceration. **A:** A patient with normal mucosa pre-ablation. **B:** Multiple ulcers with Zargar’s grade 2a damage post-ablation, with diffuse sloughing as well as multiple ulcerations, with the largest ulceration visible in the image. **C:** Another control patient with a normal esophageal mucosa pre-ablation. **D:** A large ulceration with a necrotic core in the same patient post-ablation.

All enrolled patients had normal esophageal mucosa immediately following TEE on the day prior to their ablation. One patient was incidentally noted to have Barrett’s esophagus, which required long-term monitoring. In the 3 control patients, all had multiple instillations of ice-cold water for temperature excursions of >1°C during posterior wall ablation. Of these 3 patients, 1 had no evidence of esophageal mucosal damage, 1 had diffuse sloughing of the esophageal mucosa and multiple ulcerations, and 1 had a superficial ulcer with a large clot. Example EGD images of control patient lesions are shown in **Figure 2**. Both patients with lesions were classified as Zargar grade 2a **(Table 3)**.

**Figure 3:**
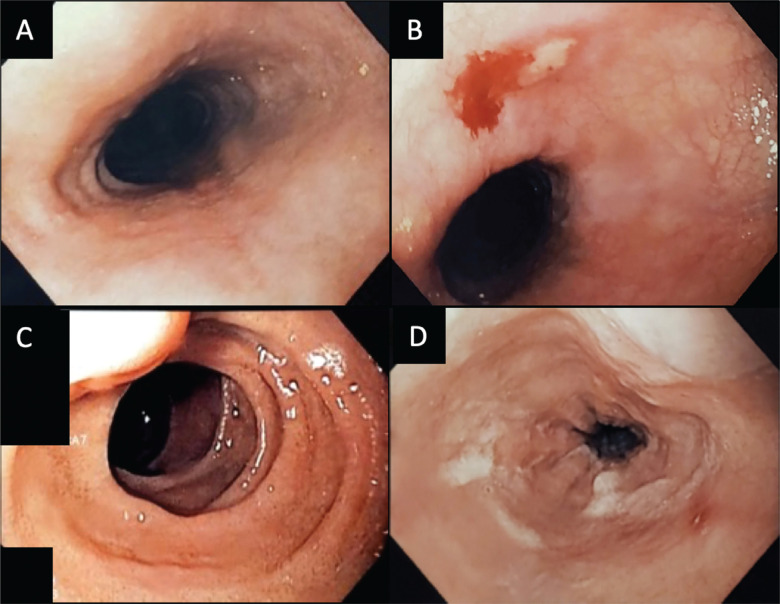
Esophagogastroduodenoscopy images of cooling catheter patients showing esophageal changes. **A:** A patient with normal mucosa pre-ablation. **B:** A patient with a post-ablation superficial ulcer (Zargar’s grade 2a). **C:** A patient with normal mucosa pre-ablation. **D:** Post-ablation erythema (Zargar’s grade 1).

In the 3 patients treated with the esophageal cooling device, 1 had no evidence of esophageal mucosal damage, 1 had esophageal erythema (Zargar grade 1), and 1 had a solitary Zargar grade 2a lesion. There was no significant difference in the total ablation time or posterior wall ablation between the groups, as seen in **Table 4**. The patient with the most extensive posterior wall ablation in the treatment group did not have any esophageal injury **(Table 3)**. Example EGD images from patients in the treatment arm are shown in **Figure 3**.

### Feasibility

The placement of the esophageal cooling device was straightforward and did not interfere with ablation, allowing the procedure to continue without interruption for temperature overshoot. Moreover, there was no need for additional fluoroscopy use as the cooling device stays placed to the depth of the stomach without the need for repositioning during the procedure.

### Patients’ follow-up results

Patients were evaluated via an event monitor or a loop recorder. Evaluations performed between 3–6 months after the ablation procedure showed recurrence of atrial fibrillation in 1 control patient and 1 treatment-arm patient.

## Discussion

This was the first randomized, controlled feasibility study of a commercially available esophageal cooling device used for esophageal protection during left atrial ablation for the treatment of atrial fibrillation. Additionally, this remains the only study to our knowledge comparing reactive cooling (in which cold water is administered through an orogastric tube into the esophagus in response to esophageal temperature rises) and proactive cooling (in which esophageal cooling is performed using a dedicated esophageal cooling device). In the latter case, esophageal cooling is attained prior to any temperature rise that would occur, which in turn negates the need to use any esophageal temperature monitoring probe. In this study, we found that the use of this device was indeed feasible for this purpose. In addition, because stopping ablation for temperature rises in the esophagus did not occur, and because repositioning of the traditionally used temperature probe was not required, an improvement in workflow was noted. A subsequent investigation into the procedural impact of active esophageal cooling has since been published, reporting a mean procedure time reduction of 36 min, or 24.7% of the total procedure time (*P* < 0.001).^20^

Although not powered for further hypothesis testing, the extent and severity of esophageal lesions in actively cooled patients were slightly less than those in control patients receiving intermittent reactive instillation of ice-cold water in response to temperature elevations. Specifically, the treatment arm cohort included a single ulcer in 1 patient and an erythematous patch in another patient. The control group included 2 patients with esophageal lesions with higher degrees of injury, diffuse sloughing, and larger ulcerations.

Various approaches have been developed to attempt esophageal protection during RF ablation, including LET monitoring, cooling, and deviating the esophagus. Nevertheless, currently available discrete sensor probes, whether single or multiple in number, do not appear to significantly reduce injury rates, and there is a potential for esophageal harm with esophageal deviation.^21–23^ In contrast, multiple earlier studies have evaluated esophageal cooling using a variety of techniques, with all but 1 suggesting a potential benefit.^6–13^ The availability now of a commercial device currently on the market for whole-body temperature modulation offers the chance to further study this modality, which, in our data, appears to offer potential. Indeed, subsequent studies comparing active esophageal cooling with this device to standard LET monitoring (without the use of reactive cooling via manual cold-water instillation) have since been performed. The Esophageal Cooling for AF Ablation (eCOOL-AF) pilot study randomized 44 patients to single-sensor LET monitoring or active esophageal cooling, finding a 67% reduction in severe esophageal lesions with active esophageal cooling.^24^ The larger confirmatory IMPACT study randomized 120 patients to single-sensor LET monitoring or active esophageal cooling, finding an 83% reduction in esophageal lesions with active esophageal cooling.^25^ Although both studies used the same active cooling device, neither study employed the reactive cooling strategy in the control arm used in our study. Reactive cooling using direct instillation of cold water into the esophagus via an orogastric tube has a limited heat extraction capacity, as small aliquots of cold water have a limited capacity to absorb heat. Nevertheless, a meta-analysis of 3 studies totaling almost 500 patients evaluating this technique for esophageal protection found an odds ratio of 0.39 (95% confidence interval, 0.17–0.89) for the reduction of severe-grade lesions, suggesting that even a technique with low heat extraction capacity may still offer benefits over LET monitoring alone.^26^ Thus, our pilot study includes a control arm, which is likely a more difficult one against which to demonstrate superiority.

### Limitations

We were not able to blind the physician performing the ablation and, as such, differences in ablation technique may have occurred. Nevertheless, because cessation of ablation occurred regularly in the control arm after elevated temperature probe readings, but did not occur in the treatment arm, it is likely that the actual local energy deposition in many areas was greater in the treatment arm. A number of other factors, such as trends toward a larger left atrial size, lower ejection fraction, more obstructive sleep apnea, and more obesity in the treatment arm, may have influenced our findings. Our standard practice also uses non–contact-force-sensing, non-irrigated catheters. As such, parameters such as the force–time integral or ablation index were not possible to measure but may have varied between groups for a variety of reasons. However, non-irrigated and non–contact-force-sensing catheters were used by 30% of the writing group of the 2017 Heart Rhythm Society/European Heart Rhythm Association/European Cardiac Arrhythmia Society/Asia Pacific Heart Rhythm Society/Latin American Society of Electrophysiology and Cardiac Stimulation Expert Consensus Statement on Catheter and Surgical Ablation of Atrial Fibrillation; therefore, generalizability is not precluded.^27^ Another limitation of this study is its sample size of 6 patients. While this smaller sample size does limit the impact of randomization, this study was planned as an initial feasibility study into the use of the device and so was not intended or powered to provide statistically significant findings. However, this study has been further referenced and built upon with larger randomized controlled trials that confirm both the originally sought determination of feasibility and the actual clinical benefits of active esophageal cooling. This study remains the first to investigate feasibility and the only to compare active esophageal cooling to reactive ice-water instillation.

## Conclusions

Although a number of subsequent studies have confirmed the reduction of esophageal injury with the use of proactive esophageal cooling, this study is the only investigation to date comparing reactive cooling (via manual cold-water instillation) and proactive cooling (via a dedicated esophageal cooling device). Moreover, this was the first study to support the feasibility of using a dedicated cooling device for this purpose and has provided the basis for further investigation.

## Figures and Tables

**Table 1:** Patient Characteristics

Characteristics	Cooling Catheter n = 3	Control Group n = 3
Age (avg) (years)	58–70 (64.7)	55–71 (61.3)
Left atrial size (avg) (cm)	4.1–5.7 (4.7)	3.8–4.2 (3.9)
Ejection fraction <50% (avg)	2 (51%)	0 (58%)
Hypertension	3	2
Coronary artery disease	1	1
Diabetes mellitus	0	1
Cerebrovascular accident	1	0
Vascular disease	0	0
Obstructive sleep apnea	2	0
Obesity	2	1

**Table 2:** Zargar Classification and Its Corresponding Endoscopic Description

Zargar Classification	Description
Grade 0	Normal mucosa
Grade 1	Edema and erythema of the mucosa
Grade 2a	Hemorrhage, erosions, blisters, superficial ulcers
Grade 2b	Circumferential lesions
Grade 3a	Focal deep gray or brownish-black ulcers
Grade 3b	Extensive deep gray or brownish-black ulcers
Grade 4	Perforation

**Table 3:** Study Results

Study Patient	Age/Sex	ECD	Indication	Zargar’s Grade Pre-/postoperation	Postoperation Ulcers	Procedure	Total Ablation Lesions	Posterior Wall Lesions	Postoperation monitoring
186-1	58/F	No	Paroxysmal AF	0/0	0	PVI with LA roof line	273	45	No AF on loop recorder
186-2	58/M	Yes	Paroxysmal AF and typical atrial flutter	0/2a	1	PVI, roof line, and CTI line	128	21	Palpitations—AF noted on event recorder at 30 days
186-3	66/M	Yes	Paroxysmal AF and atypical atrial flutter	0/0	0	PVI, roof line, mitral isthmus line, CTI line, and CFAE ablation	511	118	Palpitations—no AF or atrial flutter on event recorder
186-4	55/M	No	Paroxysmal AF	0/2a	Multiple	PVI	320	48	No AF on event monitor at 6 months
186-5	70/M	Yes	Atypical atrial flutter	0/1	0 ulcers–erythema noted	Roof line, mitral isthmus line, CTI line, and CFAE ablation	273	5	Palpitations (much improved)—no AF on event monitor
186-6	71/M	No	Paroxysmal AF	0/2a	1	PVI, roof line	511	118	AF noted on loop recorder at 30 days

*Abbreviations:* AF, atrial fibrillation; CFAE, complex fractionated atrial electrogram; CTI, cavotricuspid isthmus; ECD, esophageal cooling device; LA, left atrium. Results of all patients, including indication for procedure, esophageal findings, and postoperation monitoring, are presented. The total number of ablation lesions and the number of lesions applied to the posterior wall are also included for comparison between patients.

**Table 4:** Ablation Data Averaged for the Control and Treatment Groups

	Esophageal Cooling Device Used				*P* Value
	No		Yes		
	Mean	Standard Deviation	Mean	Standard Deviation	
Total ablation time (s)	3,700	958	3,432	1,385	1.0
Total number of RF applications	368	126	304	193	.7
Posterior wall lesions	70	41	48	61	.4
Average power (W)	34	9	31	11	1.0
Maximum power (W)	40	8	44	22	.7
Average impedance (Ω)	59	3	76	11	.1
Average temperature (°C)	46	2	47	4	1.0
Maximum temperature (°C)	56	4	60	8	.7

*Abbreviation:* RF, radiofrequency. The Mann–Whitney *U* test was used to obtain significance.
